# Clinical value of fibroblast growth factor 19 in predicting gastrointestinal dysfunction in patients with sepsis

**DOI:** 10.3389/fnut.2024.1442203

**Published:** 2024-09-04

**Authors:** Linsai Guan, Feiyao Wang, Jingni Chen, Yanxin Xu, Weixing Zhang, Jianping Zhu

**Affiliations:** ^1^Department of Nursing, Shanghai Taikang Shenyuan Rehabilitation Hospital, Shanghai, China; ^2^Department of Critical Care Medicine, Shanghai General Hospital, Shanghai Jiao Tong University School of Medicine, Shanghai, China; ^3^Department of Geriatrics, Shanghai General Hospital, Shanghai Jiao Tong University School of Medicine, Shanghai, China

**Keywords:** fibroblast growth factor 19 (FGF19), gastrointestinal dysfunction, procalcitonin, sepsis, total bile acid (TBA)

## Abstract

**Objective:**

To assess the potential value of fibroblast growth factor 19 (FGF19) as a predictor of gastrointestinal (GI) dysfunction in patients with sepsis.

**Methods:**

A prospective study was conducted, and 209 patients who were diagnosed with sepsis and admitted to the intensive care unit (ICU) at teaching hospitals in China were enrolled from June 2023 to December 2023. The serum FGF19 level was determined at ICU admission. The differences in serum FGF19 levels between the two groups were compared via the Mann–Whitney U test, and Spearman’s correlation coefficient was used to identify the correlations of the FGF19 concentration with other clinical variables and biomarkers. Receiver operating characteristic (ROC) analysis was used to determine the value of FGF19 in predicting GI dysfunction in patients with sepsis.

**Results:**

The total ICU mortality rate was 13.3% (24/180). There was a tendency toward increased ICU mortality in patients with sepsis-associated GI dysfunction compared with patients without GI dysfunction with statistical significance (21.9% vs. 8.6%, *p* = 0.031). Serum FGF19 levels were significantly higher in patients with sepsis-associated GI dysfunction than in patients without GI dysfunction [355.1 (37.2, 2315.4) μg/mL vs. 127.4 (5.7, 944.2) μg/mL, *p* = 0.003]. The results of receiver operating characteristic (ROC) curve analysis revealed that the area under the ROC curve (AUC) for the ability of FGF19 to predict GI dysfunction in patients with sepsis was 0.773 (95% CI 0.712 ~ 0.827), which was greater than the predictive capacity of PCT [AUC = 0.632 (95% CI 0.562 ~ 0.804)].

**Conclusion:**

Serum FGF19 could be considered as a novel predictor or biomarker of GI dysfunction in patients with sepsis.

## 1 Introduction

Sepsis is a common critical disease in intensive care units (ICUs), and its incidence is related to the body’s response to infection, with the progression of the disease leading to multiple organ dysfunction. Severe cases can be life-threatening, however, in recent years, the diagnosis and treatment of sepsis have made great progress, but the mortality rate of patients is still as high as 30% (1–3). Acute gastrointestinal (GI) dysfunction (4) is an acute pathological change in the GI tract secondary to trauma, burn, shock and other systemic lesions, with GI mucosal damage as well as motor and barrier dysfunction as its main features. The disease is not a separate group of diseases but rather a descriptively elusive component of multiple organ dysfunction syndrome. These include acute gastric mucosal lesions, acute non-calculous cholecystitis, translocation of the intestinal flora and toxins, diarrhea associated with critical illness, and slow or disappearance of intestinal peristalsis caused by nerve palsy. Owing to its unclear definition and classification, the Sequential Organ Failure Assessment (SOFA) score was not included. However, studies have shown that GI dysfunction occurs in approximately 85% (470/550) of patients in the ICU and is closely related to prognosis, which has become a weak link in the study of multiple organ dysfunction (5). Therefore, monitoring, prevention and treatment of GI dysfunction in critically ill patients have received increasing attention from medical personnel (6, 7).

At present, there are no effective predictors of GI dysfunction in sepsis patients. The acute GI dysfunction classification proposed by the European Society of Intensive Care Medicine (ESICM) in 2012 is susceptible to subjective factors, and its clinical application is limited (8, 9). Therefore, the development of early GI dysfunction predictive biomarkers has important clinical value. During fetal life, fibroblast growth factor (FGF)15/19 is involved in organogenesis, affecting the development of the ear, eye, heart, and brain. In adulthood, FGF15/19 is produced mainly by the ileum and acts on the liver to repress hepatic bile acid synthesis and promote postprandial nutrient partitioning (10). Lee et al. (11) reported that the secretion of FGF19 prevented the excessive production of intestinal bacteria and their entry into the portal vein, and inhibited the progression of liver inflammation. Zhao et al. (12) reported that FGF19 can inhibit the NF-κB signaling pathway to achieve anti-inflammatory effects, which is a key feature in the early stage of sepsis, and that the FGF19 may be a predictive biomarker of GI dysfunction. Therefore, increased FGF19 levels might be an early predictor of acute gastrointestinal (AGI) dysfunction in sepsis patients. Accordingly, we hypothesized that increased FGF19 levels are associated with AGI development in sepsis patients, and the aim of this manuscript is performed to verify this hypothesis.

In this study, sepsis patients in the ICU of the Shanghai General Hospital from June 2023 to December 2023 were included to observe serum FGF19 level and explore the relationship between serum FGF19 level and GI dysfunction. To provide a reference for the prediction of sepsis complicated with GI dysfunction.

## 2 Methods

### 2.1 Patients

**Figure 1:**
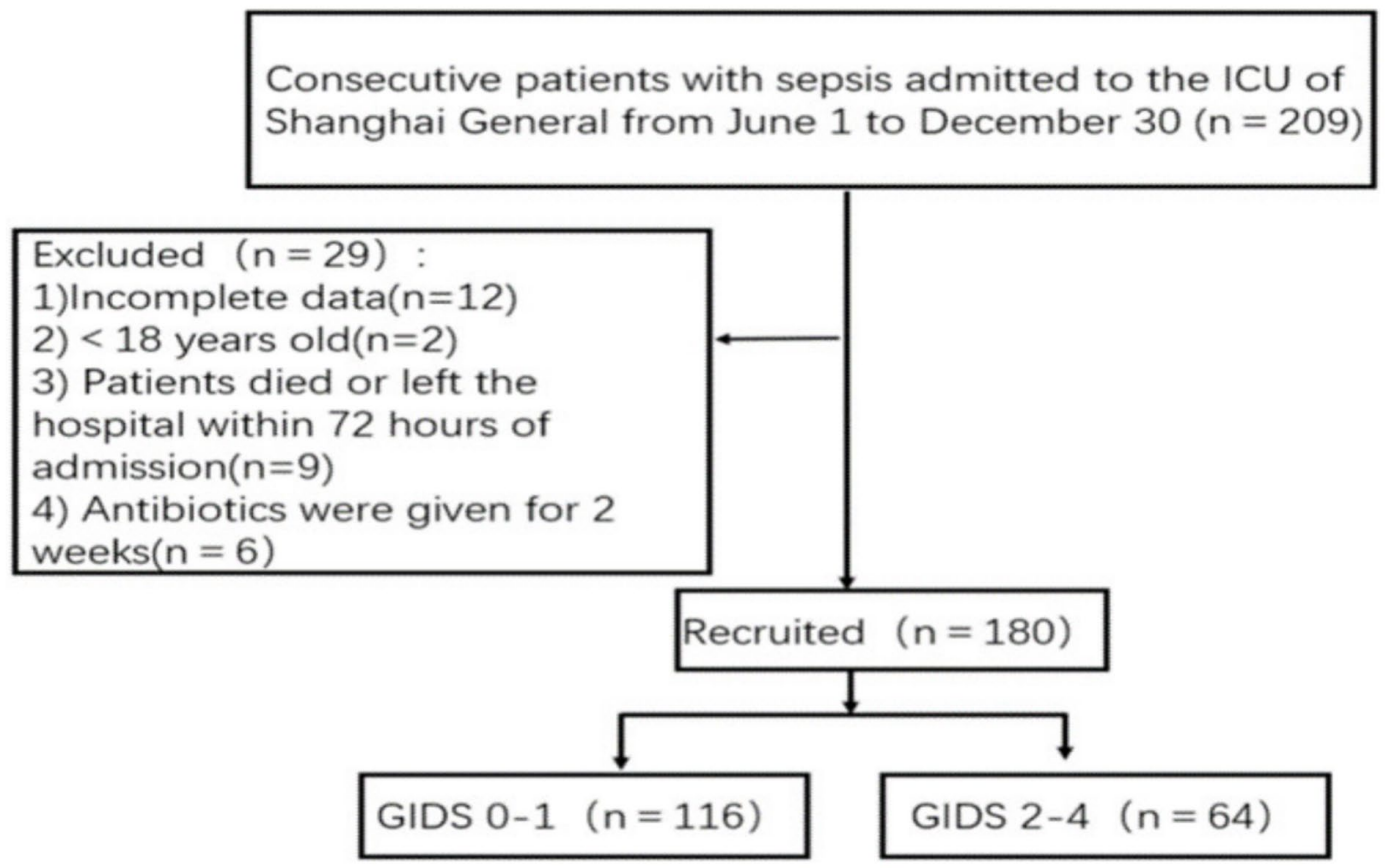
The flow of patient selection. GIDS, Gastrointestinal Dysfunction Score.

This prospective observational study recruited continuous patients who were diagnosed with sepsis and admitted to the ICUs of teaching hospitals in China (Class III, Class A hospital) from June 1, 2023, to December 30, 2023; these patients were divided into a sepsis group without GI dysfunction and a sepsis group with GI dysfunction according to their presence or absence (Figure 1). Each patient was followed for 28 days, and the last follow-up for all patients was completed on January 30, 2024. All enrolled patients received standard care during their intensive care unit (ICU) stay. This study was approved by the ethics committee of Shanghai General Hospital (2,023, 190). Each procedure adhered to the Helsinki Declaration. Informed consent and signatures were obtained from all patients or their guardians.

The inclusion criteria for patients were as follows: met the diagnostic criteria for sepsis and the SOFA score defined in the Third International Consensus on the Definition of Sepsis and Septic Shock (Sepsis 3.0) (13) (suspected clinical infection and SOFA score ≥ 2); all patients were ≥ 18 years old. The exclusion criteria were as follows:(1) patients who had used antibiotics in the past two weeks; (2) patients who were clearly diagnosed with malignant tumors; (3) patients who died within 72 h after admission; (4) patients whose medical records were incomplete. According to Gastrointestinal Dysfunction Score (GIDS), the patients were divided into two groups: the sepsis without GI dysfunction group and the sepsis with GI dysfunction group.

### 2.2 Data collection

A case report form (CRF) was established in advance, and clinical parameters such as age, sex, body mass index (BMI), past medical history, source of infection, and mechanical ventilation were recorded. The laboratory indicators included the following: ① Routine blood indicators, including white blood cells (WBCs) and platelets (PLTs). ② Indicators related to organ function, including total bilirubin (TBIL), albumin (ALB), total bile acid (TBA), blood urea nitrogen (BUN), creatinine (Cr), and lactate (Lac) levels. ③ Coagulation function indicators, including activated partial thromboplastin time (APTT), the international normalized ratio (INR), and fibrinogen (Fib). ④ Susceptibility indicators, including PCT and C-reactive protein (CRP), were measured. The outcome variables included length of ICU stay and ICU mortality. The experimental indicators were collected within 24 h after ICU admission. The GIDS, Acute Physiology and Chronic Health Evaluation II (APACHE II), and SOFA scores were recorded daily for the first 7 days after ICU admission. FGF19 levels were measured twice within 3 days after admission and recorded. Take the average of the two measured values.

The worst GIDS score within 7 days after admission, the number of patients receiving total enteral nutrition and the number of patients with feeding intolerance were recorded. The primary end point was the ratio of GI deterioration, and the secondary end points were the feeding intolerance incidence rates and total enteral nutrition ratios within 7 days after admission. To ensure the accuracy of the data, two specially trained medical staff members are responsible for collecting data from all patients. The patient data collected by the night doctor were verified for eligibility by two specially trained medical staff.

Measurement of markers: At the time of admission to the ICU, peripheral venous blood was collected under non-anticoagulant conditions before antibiotics were administered. Serum was collected after centrifugation and stored at −80°C for future use. Serum FGF19 levels were determined via an enzyme-linked immunosorbent assay (ELISA) kit (Hangzhou Lianke Biotechnology Co., Ltd.).

### 2.3 Gastrointestinal dysfunction score (GIDS) evaluation

**Figure 2:**
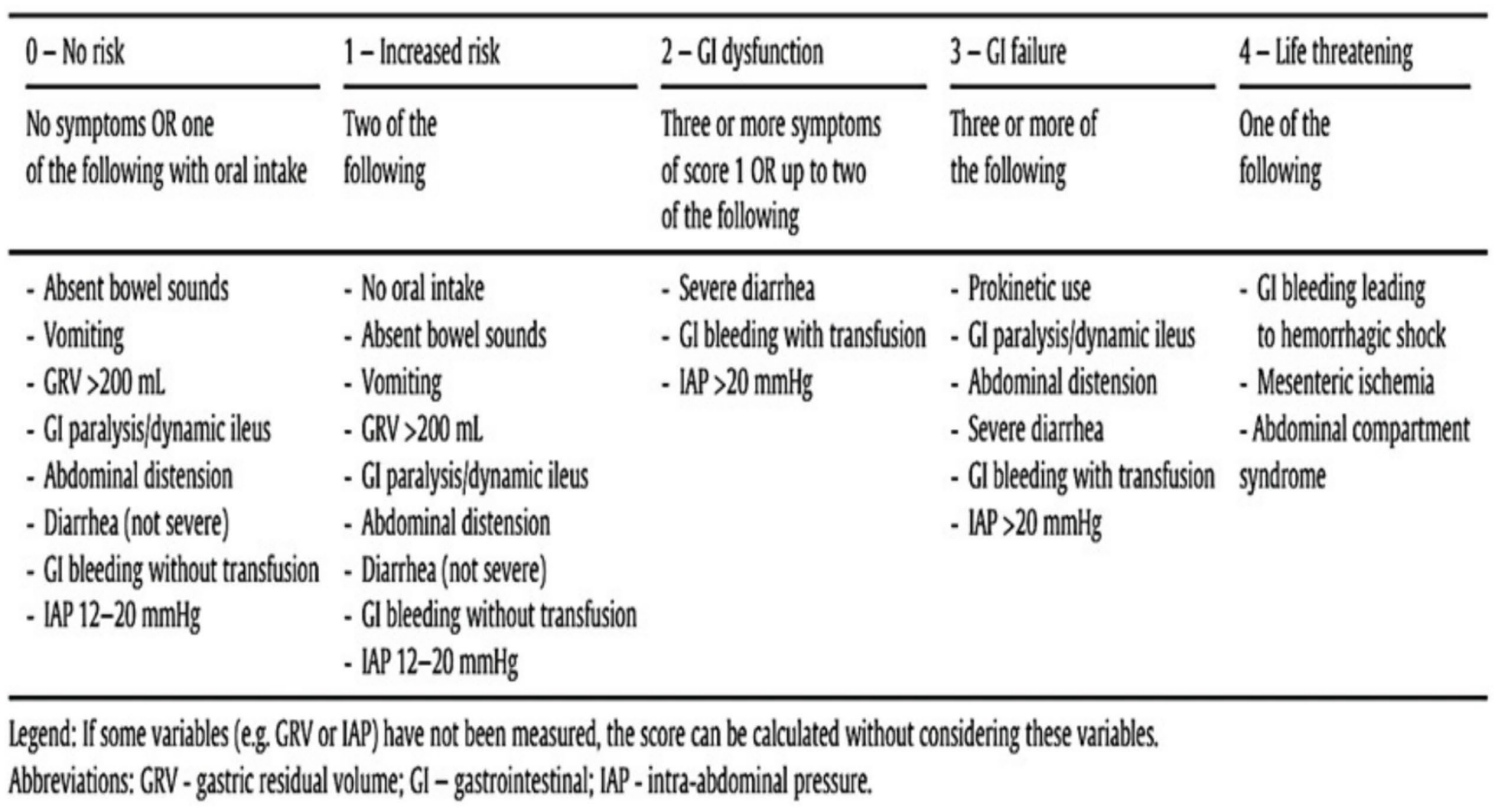
GIDS (Gastrointestinal Dysfunction Score).

The GIDS, a new gastrointestinal dysfunction score developed by Blaser et al. (14) for critically ill patients, was applied in this study. The scoring system was divided into 5 grades (Figure 2). Liu et al. (9) studied and verified a GIDS and achieved the expected effect. Prior to the start of the study, two medical staff members were trained in the scoring of the GIDS scale. Daily GI and abdominal symptoms (vomiting/reflux, loss of intestinal sounds, diarrhea, bloating, GI bleeding, GI paralysis/dynamic ileus), gastric residual volume (GRV), intra-abdominal pressure (IAP), nutritional, and GI medication data were recorded for patients with GI dysfunction after admission. All variables are defined as recommended by international consensus. Severe diarrhea (Bristol grade 6–7) is defined as the presence of 1,000 mL of stool per day or five times a day. The IAP and GRV were measured in patients by indwelling bladder catheters or nasogastric tubes. On the basis of acute gastrointestinal injury (AGI) scoring experience (divided into groups І-II and III-IV), the patients in this study were divided into a group without GI dysfunction (0–1) and a group with GI dysfunction (2–4) according to the GIDS.

### 2.4 Statistical analysis

Statistical analysis was performed via STATA 15.0MP (College Station, Texas, United States) software. The Kolmogorov–Smirnov test was used for normality analysis of the quantitative data. Quantitative data following a normal distribution are presented as x̄ ± s, and differences between groups were compared via *t* tests. Quantitative data with a non-normal distribution are expressed as M (Q_1_, Q_3_), and nonparametric rank sum tests were used to compare differences between groups. Qualitative data are expressed as *n* (%), and differences between groups were compared via the *χ*^2^ test. Spearman correlation coefficients were used to determine the associations of the FGF19 concentration with other clinical variables and biomarkers. Receiver operating characteristic (ROC) curves were used to analyze and evaluate the predictive efficacy of FGF19 and PCT in sepsis patients complicated with GI dysfunction. *p* < 0.05 indicated that the difference was statistically significant.

## 3 Results

### 3.1 Patient characteristics

A total of 180 patients with sepsis were included, including 102 males and 78 females. Patients were divided into two groups according to the presence or absence of GI dysfunction: the sepsis without GI dysfunction group (*n* = 116) and the sepsis with GI dysfunction group (*n* = 64). In 64 patients with sepsis complicated with GI dysfunction, the interval time from ICU admission to GI dysfunction was 0 (0, 4.0) days, and the duration of GI dysfunction during the ICU stay was 4.0 (3.0, 8.0) days. There were significant differences in the SOFA score and APACHE II score between the two groups (*p* < 0.05). The mortality rate of sepsis in the ICU was 13.3% (24/180). The mortality rate of patients with sepsis combined with GI dysfunction in the ICU was significantly greater than that of patients with sepsis without GI dysfunction (21.9% vs. 8.6%) (*p* = 0.031). There were no statistically significant differences in age, sex, BMI, previous history, source of infection, diabetes, hypertension or mechanical ventilation between the two groups (Table 1).

**Table 1:** Baseline characteristics of patients with sepsis.

Parameter	Sepsis without GI dysfunction (*n* = 116)	Sepsis with GI dysfunction (*n* = 64)	*χ*^2^/*t*/*Z*	*p* value
Male sex	62 (53.4%)	40 (62.5%)	1.376	0.241
Age, years	66.07 ± 12.13	66.82 ± 14.07	1.119	0.292
Body mass index, kg/m^2^	23.16 ± 2.07	23.72 ± 2.46	1.177	0.242
SOFA	8.32 ± 2.78	11.12 ± 3.18	4.638	<0.001
APACHEII	14.32 ± 3.15	23.68 ± 5.69	10.625	<0.001
Type 2 diabetes	44 (37.9%)	39 (60.9%)	3.116	0.078
Hypertension	64 (55.2)	43 (67.2%)	2.469	0.116
Septic shock	–	–	3.807	0.051
Yes	26 (22.4%)	23 (35.9%)	–	–
No	90 (77.6%)	41 (64.1%)	–	–
Infection site, *n* (%)	–	–	7.416	0.116
Respiratory system	57 (49.1)	24 (37.5)	–	–
Abdominal infection	26 (22.4)	25 (39.1)	–	–
Urine system	4 (3.4)	1 (1.6)	–	–
Nervous system	14 (12.1)	4 (6.3)	–	–
Others	15 (12.9)	10 (15.6)	–	–
AKI stage	–	–	1.089	0.580
Stage І	10 (8.6%)	21 (32.8%)	–	–
Stage II	7 (6.0%)	16 (25.0%)	–	–
Stage III	14 (12.1%)	19 (29.7%)	–	–
Mechanical ventilator, *n* (%)	65 (56.0)	44 (68.8)	2.792	0.09
Hospital length of stay, days	21.4 (14.7, 36.9)	23.6 (15.6, 38.2)	−0.249	0.804
ICU mortality, *n* (%)	10 (8.6)	14 (21.9)	4.652	0.031

GI, gastrointestinal; APACHE II, Acute Physiology and Chronic Health Evaluation II; SOFA, sequential organ failure assessment.

### 3.2 Relationships between serum laboratory indices and GI dysfunction in sepsis patients

The serum FGF19 level of sepsis patients with GI dysfunction was significantly greater than that of sepsis patients without GI dysfunction when admitted to the ICU [355.1 (37.2, 2315.4) pg./mL vs. 127.4 (5.7, 944.2) pg./mL, *p* = 0.003]. Compared with that in the sepsis group without GI dysfunction, the PCT level was significantly greater in the sepsis group with GI dysfunction [7.12 (5.12, 8.13) ng/mL vs. 3.71 (3.29, 4.23) ng/mL, *p* < 0.001]. Compared with that in the sepsis group without GI dysfunction, the TBA level was significantly greater in the sepsis group with GI dysfunction [15.26 ± 2.05 (μmol/L) vs. 9.88 ± 1.32 (μmol/L), *p* < 0.001]. The level of serum Lac in patients with sepsis combined with GI dysfunction tended to increase, but the difference was not statistically significant (*p* = 0.481). There was no significant difference in WBC, PLT, TBIL, ALB, BUN, Cr, APTT, INR, Fib or CRP between the two groups (Table 2).

**Table 2:** Comparison of the laboratory indices of septic patients with GI dysfunction and without GI dysfunction.

Parameter	Sepsis without GI dysfunction (*n* = 116)	Sepsis with GI dysfunction (*n* = 64)	*χ*^2^/*t*/*Z*	*P* value
FGF19(pg/mL)	127.4 (5.7, 944.2)	355.1 (37.2, 2315.4)	−2.933	0.003
WBC(10^9^/L)	12.7 (2.0, 54.2)	15.0 (1.2, 32.2)	0.198	0.847
PLT(10^9^/L)	135.00 (101.00, 184.00)	146.00 (89.00, 213.00)	−1.678	0.093
INR	1.20 (1.10, 1.40)	1.40 (1.20, 1.80)	−0.249	0.804
APTT(s)	29.10 (26.20, 33.20)	32.20 (27.45, 38.80)	1.600	0.113
Fib(g/L)	2.2 (1.4, 4.1)	2.6 (1.7, 3.9)	−1.135	0.256
ALB(g/L)	30.35 (27.58, 33.93)	32.75(28.85, 35.98)	−1.365	0.172
TBIL (μmol/L)	14.8 (4.5, 119.2)	23.7 (3.9, 124.7)	0.606	0.546
TBA(μmol/L)	9.88 ± 1.32	15.26 ± 2.05	14.422	<0.001
Cr (μmol/L)	274.96 ± 17.88	279.52 ± 19.23	1.148	0.254
BUN (mmol/L)	15.26 ± 2.93	16.24 ± 2.31	1.631	0.106
LAC(mmol/L)	2.20 (1.05, 6.20)	4.20 (2.80, 8.50)	0.890	0.373
PCT(ng/mL)	3.71 (3.29, 4.23)	7.12 (5.12, 8.13)	−4.815	<0.001
CRP(mg/L)	122.12 ± 86.61	145.28 ± 94.45	−1.152	0.253

WBC, white blood cell; PLT, platelet; TBIL, total bilirubin; ALB, albumin; BUN, blood urea nitrogen; Cr, creatinine; APTT, activated partial thromboplastin time; INR, international normalized ratio; Fib, fibrinogen, CRP, and reactive protein. PCT, procalcitonin; TBA, total bile acid.

### 3.3 Correlation analysis between FGF19 and the APACHE II score, SOFA score and PCT

In this study, the correlation analysis of FGF19 with the APACHE II score, SOFA score and PCT score revealed that the level of FGF19 was positively correlated with the APACHE II score, SOFA score and PCT score [(*r* = 0.503, *p* < 0.001), (*r* = 0.471, *p* < 0.001), (*r* = 0.416, *p* < 0.001)] (Table 3).

**Table 3:** Correlations of FGF19 levels with different clinical parameters and blood markers in sepsis patients.

Variable	Pearson’s coefficient	*P* value
APACHEII	0.503	<0.001
SOFA	0.471	<0.001
TBA	0.817	<0.001
PCT	0.416	<0.001

APACHE II, Acute Physiology and Chronic Health Evaluation II; SOFA, sequential organ failure assessment; PCT, procalcitonin; TBA, total bile acid.

### 3.4 Serum FGF19 as a predictor of GI dysfunction in sepsis patients

**Figure 3:**
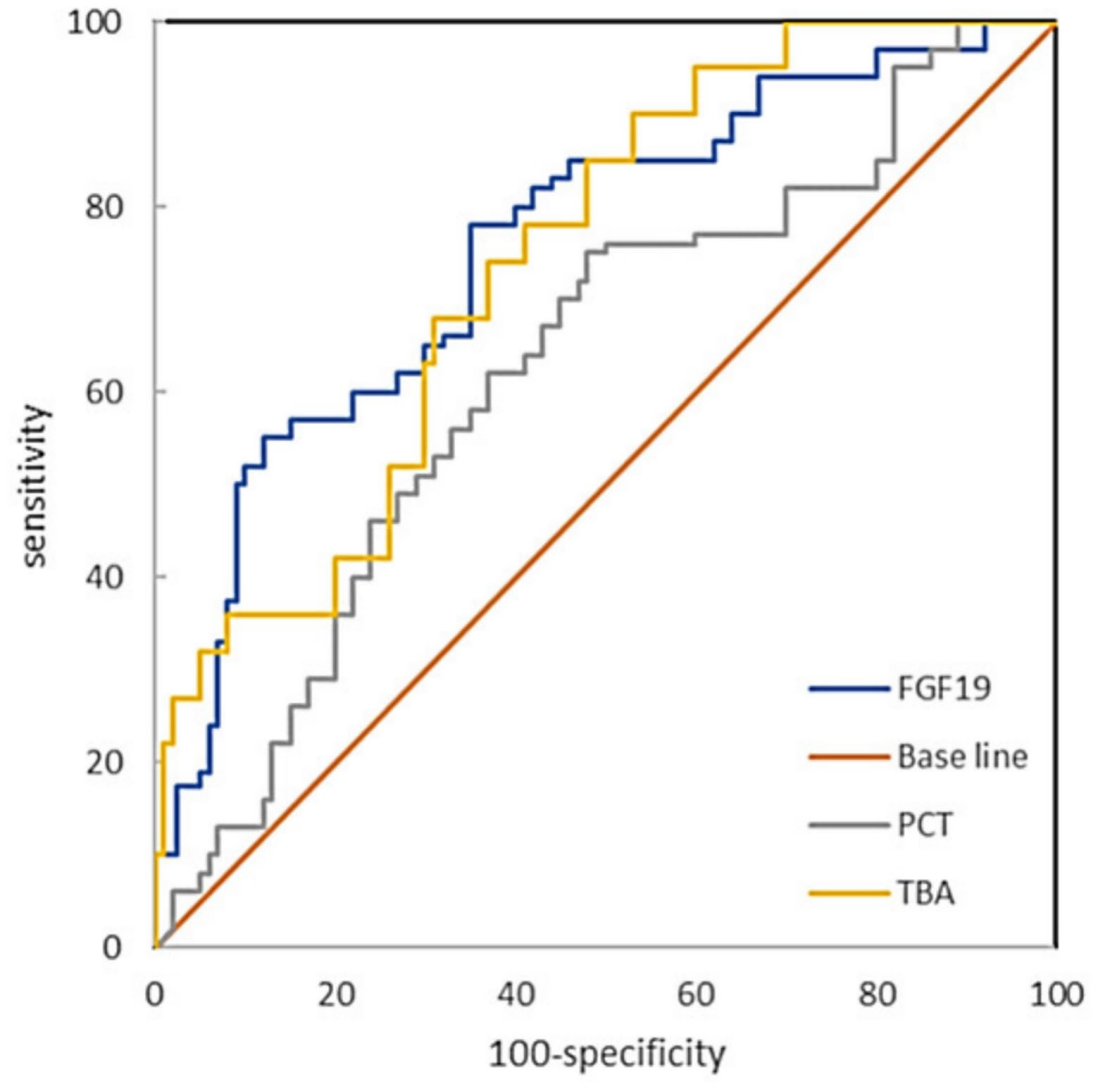
ROC curve analysis of the ability of serum FGF19 and PCTlevels to predict GI dysfunction in patients with sepsis.

ROC curve analysis results showed that the area under ROC curve (AUC) of FGF19 in predicting sepsis complicated with GI dysfunction was 0.773 (95%CI 0.712–0.827). The AUC corresponding to PCT was 0.632 (95%CI 0.562 ~ 0.804). When the cutoff value of serum FGF19 was 210 μg/mL, the sensitivity and specificity of predicting sepsis with GI dysfunction were 78.3 and 65.3%, respectively. When the cutoff value of serum PCT level was 5.31 ng/mL, the sensitivity was 60.0%, and the specificity was 66.5% (Table 4, Figure 3).

**Table 4:** Efficacy analysis of serum FGF19 and PCT in predicting GI dysfunction in patients with sepsis.

Variable	Youden index	Cut-off value	AUC	95%CI	Sensitivity (%)	Specificity (%)
FGF19	0.436	210 pg./mL	0.773	0.712 ~ 0.827	78.3	65.3
PCT	0.265	5.31 ng/mL	0.632	0.562 ~ 0.804	60.0	66.5
TBA	0.378	11.63	0.759	0.704 ~ 0.810	75.6	62.2

AUC, area under the ROC curve; TBA, total bile acid; PCT, procalcitonin; FGF19, fibroblast growth factor 19.

## 4 Discussion

In the intensive care setting, GI dysfunction is a common complication among critically ill patients and is closely linked to patient outcomes (15, 16). In this study, the incidence of GI dysfunction in patients with sepsis was 35.6% (64/180). The ICU fatality rate of sepsis patients with GI dysfunction was 21.9%, which was significantly greater than that of patients without GI dysfunction (8.6%) (*p* = 0.031). Early identification of sepsis complicated with GI dysfunction and timely intervention are key to improving the prognosis of sepsis patients.

The serum PCT level has important clinical value in sepsis infection and related organ damage (17, 18). Studies have shown that PCT has clinical significance in the diagnosis of intestinal ischemia, early prediction and timely diagnosis and treatment of inflammatory bowel disease (19, 20). These findings suggest that sepsis complicated with GI dysfunction may be closely related to changes in the serum PCT level. In this study, the serum PCT level was used to predict sepsis complicated with GI dysfunction. When the cutoff value was 5.31 ng/mL, the AUC was 0.632, the sensitivity was 60.0%, and the specificity was 66.5%. These findings suggest that PCT is an effective biomarker for predicting sepsis complicated with GI dysfunction. Since PCT is a routine clinical parameter, its clinical importance in predicting and identifying sepsis complicated with GI dysfunction deserves further attention.

This study revealed that in the early stage of sepsis, the serum FGF19 concentration in sepsis patients with GI dysfunction was almost three times greater than that in sepsis patients without GI dysfunction. When the cutoff value of serum FGF19 was 210 μg/mL, the corresponding sensitivity was 78.3% and the specificity was 65.3%, suggesting that an increase in the serum FGF19 concentration was a significant predictor of sepsis combined with GI dysfunction. This study also revealed that the level of TBA in sepsis patients with GI dysfunction was significantly greater than that in sepsis patients without GI dysfunction. The AUC for predicting GI dysfunction in sepsis patients was 0.759, with a sensitivity of 75.6% and a specificity of 62.2%. Shi et al. (21) reported a relationship between bile acid levels and GI dysfunction, but thea relationship between sepsis combined with GI dysfunction and bile acid levels has not been reported. Many studies have shown that TBA and FGF19 are closely related (22–24). Schaap et al. (25) reported that FGF19 was highly expressed in extrahepatic cholestasis. Cholestasis is a common complication of sepsis, and increased plasma levels of bile acids are predictive of sepsis-associated mortality. However, the exact mechanism by which cholestasis aggravates sepsis development remains elusive (26). Sun et al. (27) suggested that bile acid activates farnesoid X receptor (FXR) in the intestine and induces the expression of fibroblast growth factor 15 (FGF15, homologous with human FGF19) when patients with sepsis have abnormal liver function. FGF15 decreases bile acid levels through a gut–microbiota–liver feedback loop by suppressing the expression of cholesterol 7α-hydroxylase (CYP7A1) in the liver—the rate-limiting step in bile acid synthesis. In the FGF metabolic axis (28), FGF19 is responsible for the negative feedback regulation of bile acid synthesis. However, in this study, both bile acid and FGF19 levels were elevated in sepsis patients with GI dysfunction, indicating that FGF metabolic axis did not play a decisive role in the changes of bile acid and FGF19 levels in patients with sepsis. The main manifestations of septic liver injury are hypoxic hepatitis caused by ischemia, cholestatic liver injury caused by bile metabolism disorders, and liver injury caused by an excessive inflammatory immune response (29). Leonhardt et al. (30) reported that circulating bile acids capable of inducing immunosuppression are present in septic shock patients with severe liver failure. Chen et al. (31) reported that intestinal FGF19 secretion and associated inhibition of hepatic CYP7A1 expression provided evidence of physiologically relevant gut–liver crosstalk. The excessive increase in FGF19 may involve the disruption of bile acid homeostasis, carbohydrate metabolism, protein synthesis, lipid metabolism and glucose metabolism (32–36). We believe that the apparent increase in FGF19 in sepsis patients with GI dysfunction may be due to Bile acid regulation failure and increased responsiveness to inflammation (12), and the specific mechanisms need to be further studied. This study also revealed that the FGF19 level was positively correlated with the SOFA score and APACHE II score in sepsis patients with GI dysfunction. Li et al. (37) reported that the level of FGF19 in deceased sepsis patients was significantly greater than that in non-deceased patients. These findings suggest that the concentration of FGF19 may reflect the severity of sepsis. In addition, the results of this study revealed that baseline serum concentrations of FGF19 were positively correlated with baseline concentrations of PCT, a typical inflammatory biomarker for sepsis. These results suggest that the level of FGF19, an anti-inflammatory cytokine, increases simultaneously with that of proinflammatory cytokines in the early stages of sepsis. Since FGF19 is an intestinal secreted protein, compared with PCT which is an indicator of infection, it is speculated that FGF19 has the advantage of indicating intestinal lesions. In this study, FGF19 was associated with abnormal GI function in patients with sepsis, which provides a new perspective for the assessment of GI dysfunction.

### 4.1 Limitations

There are some limitations in this study. First of all, as a reference biomarker for GI dysfunction, the sample size is small due to the limitation of blood sample size, and there may be bias. Second, there was no subgroup analysis of the severity of GI dysfunction and the source of infection in patients with sepsis combined with GI dysfunction. Third, this study did not analyze the dynamic changes of FGF19. Despite the above research limitations, FGF19, as a specific intestinal secreted protein, is closely related to GI dysfunction associated with sepsis, and is expected to become a novel biomarker for predicting GI dysfunction.

## 5 Conclusion

In conclusion, serum FGF19 is a risk factor for sepsis combined with GI dysfunction, and the serum FGF19 level at ICU admission can be used as a novel biomarker to predict and evaluate the risk of GI dysfunction in sepsis patients during their ICU stay. Serum FGF19 concentrations above 210 μg/mL indicate an increased risk of GI dysfunction in patients with sepsis.

## Data Availability

The original contributions presented in the study are included in the article/supplementary material, further inquiries can be directed to the corresponding author.
